# 

**Published:** 1906-01

**Authors:** 


THE AMERICAN

### Journal of Dental Science

Founded, Owned, Edited and Published by the Dental Profession of America.

THIRD SERIES. D 224

VOLUME XXXVII. JANUARY, 1906. NO. 1.

EDITORS:

WM. GIRD BEECROFT, D. D. S., Madison, Wis. B. H. TEAGUE, D. D. S., Aiken, S. C.

Published on the 10th day of each month.
Subscription Price, $1 00 per year. Foreign countries, $1.50 per year.

*Entered as Second-Class Matter, Madison, Wis.*

Address all communications, both business and editorial, to
The Dental Publishing Company, Madison, Wisconsin.

